# Tunable bound states in the continuum through hybridization of 1D and 2D metasurfaces

**DOI:** 10.1515/nanoph-2025-0432

**Published:** 2025-11-10

**Authors:** Fedor Kovalev, Mariusz Martyniuk, Andrey Miroshnichenko, Ilya Shadrivov

**Affiliations:** ARC Centre of Excellence for Transformative Meta-Optical Systems (TMOS), Research School of Physics, The Australian National University, Canberra, ACT 2601, Australia; ARC Centre of Excellence for Transformative Meta-Optical Systems (TMOS), School of Engineering, The University of Western Australia, Perth, WA 6009, Australia; School of Engineering and Technology, University of New South Wales Canberra, Canberra, ACT 2612, Australia

**Keywords:** tunable metasurfaces, active metasurfaces, reconfigurable metasurfaces, all-dielectric metasurfaces, bound states in the continuum, microelectromechanical systems

## Abstract

This work presents a novel approach to create and dynamically control quasi-bound states in the continuum (BIC) resonances through the hybridization of 1D and 2D metasurfaces using micro-electromechanical systems (MEMS). The quasi-BIC resonance’s central wavelength and quality factor are precisely tuned by introducing out-of-plane symmetry breaking through a silicon MEMS membrane positioned above a 1D silicon metasurface. The proposed design achieves ultranarrow resonance linewidths with the spectral tuning range exceeding 60 nm while maintaining a constant quality factor. This tuning capability, realized through both horizontal displacement within a 1D metasurface and vertical MEMS membrane movement, offers a new degree of freedom for manipulating quasi-BIC resonances. The proposed hybridization of 2D and 1D metasurfaces using a MEMS mechanism provides a practical route to dynamic modulation of transmission resonance characteristics, making it a promising candidate for tunable filters, spectroscopy, imaging, and sensing applications.

## 1 Introduction

The concept of bound states in the continuum (BICs) represents a remarkable phenomenon in modern physics, where certain states remain localized within a system despite existing at energies or frequencies that typically allow for radiation [1]. Originating from quantum mechanics, BICs have found significant applications in photonics and metamaterials, enabling extreme confinement of electromagnetic waves and forming extremely high-*Q* resonances [2], [3]. These unique properties of BICs make them invaluable for enhancing light–matter interactions, paving the way for developing advanced photonic devices [4].

Recent advancements have focused on the active control of BICs [5], where external stimuli, such as electric fields, temperature variations, or material phase transitions, are used to dynamically tune their properties [6]. This capability enables real-time reconfiguration [7], opening up opportunities for a wide range of applications, including tunable filters [8], [9], sensors [10], [11], [12], [13], [14], and modulators [15], [16], [17], which are critical components in modern communication, imaging, and sensing technologies. The precise manipulation of quasi-BIC (q-BIC) resonances in real-time could revolutionize photonic systems, enhancing device performance and functionality, and making this a rapidly growing field in applied physics and engineering [18].

Due to their compactness, precise control, and low power consumption, micro- and nano-electromechanical systems (MEMS/NEMS) have emerged as a leading technology in fields such as active nanophotonics, sensing, and telecommunications [19], [20], [21], [22]. These systems offer dynamic, strong on-demand tuning of optical properties, which has advanced many applications by enabling integration into smaller, more efficient devices. One of the most significant breakthroughs in this area is the control of high-*Q* resonances in metasurfaces through mechanical actuation [23], [24], [25]. This has led to significant advancements, particularly in imaging and active photonics, allowing for the real-time manipulation of optical properties with minimal energy input [26], [27].

Recently, the control of guided-mode resonances (GMR) using MEMS/NEMS has opened new avenues for dynamic manipulation of light in nanophotonic devices [28], [29], [30], [31]. By integrating MEMS/NEMS, the mechanical actuation of these structures allows for precise, real-time tuning of resonance characteristics, enabling modulation of amplitude, phase, and wavelength. Notably, NEMS technology has been employed to tune both GMR and q-BIC resonances in all-dielectric metasurfaces, enabling dynamic modulation of both amplitude and phase [32]. The interference between these two resonant modes significantly enhanced the phase response of the metasurfaces, allowing for precise control with minimal input. In particular, a phase shift of 144° was achieved experimentally with just a 4 V bias change. However, this approach exhibited non-zero reflection at the q-BIC resonance, which is less than ideal for imaging applications that require minimal reflection for optimal performance. Exploring the q-BIC resonances in transmission is critical for applications such as spectroscopy, sensing and imaging, where high transmission and precise control of resonances are essential for accurate data acquisition [21].

More recently, static and thermally tunable BIC-based metasurfaces have been proposed for use in spectrometers, achieving sub-nm resolution [33], [34]. While the proposed static designs in the optical range rely on bandstop filtering and require multiple metasurfaces to cover the desired spectral range, leading to extra computations and increased spectrometer size, respectively, the designed ultracompact tunable solutions in the infrared range face limitations, including the inability to maintain a constant bandwidth and a relatively narrow spectral tuning range of only 6 nm.

This work introduces MEMS-enabled comprehensive control over q-BIC resonances in a transmission configuration within all-silicon metasurfaces. The q-BIC resonance is achieved via breaking the out-of-plane symmetry by combining a 2D MEMS structure that moves close to a 1D metasurface without direct contact. Our modeling results show that the resonance quality factor approaches infinity as the MEMS structure moves farther from a 1D metasurface, thereby reducing the asymmetry – a defining characteristic of q-BIC resonances. The vertical movement of a MEMS structure combined with horizontal displacement within a 1D metasurface enables the central wavelength to be shifted by more than 60 nm while maintaining a consistent resonance bandwidth and *Q*-factor. The proposed all-silicon metasurfaces achieve an ultranarrow resonance linewidth with dynamic tunability in the infrared range, making them promising candidates for advanced tunable filtering applications and highly relevant for spectroscopy, remote sensing, and imaging.

## 2 Results

**Figure 1:**
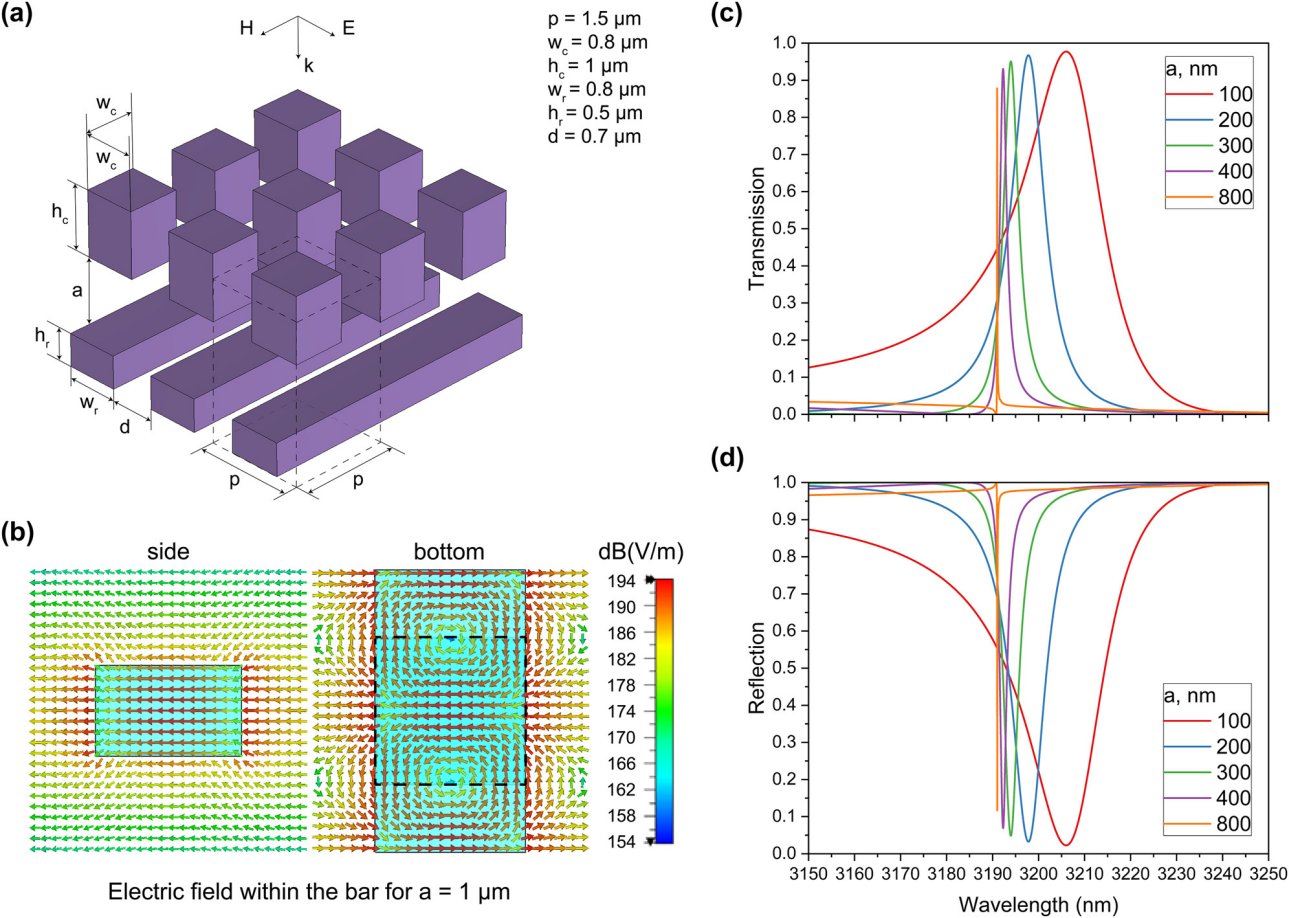
Metasurfaces based on vertically adjustable cuboids. (a) Schematic of the tunable metasurface conceptual design: silicon bars and an array of cuboids, including the unit cell indicated by the dashed lines; (b) maximum electric field amplitude of the q-BIC mode within the bar for *a* = 1 µm (side and bottom views, the dashed lines show the position of the cube); (c) and (d) transmission and reflection coefficients for various distances between silicon bars and cuboids (*a*).

We start by investigating high-*Q* q-BIC resonances excited in all-silicon metasurfaces by a plane wave under normal incidence. Figure 1(a) presents a conceptual design of a resonant structure composed of a 1D metasurface made of silicon bars and an overlying 2D array of cuboids. The structural parameters shown in Figure 1(a) are as follows: *w*_*c*_ = 0.8 µm, *h*_*c*_ = 1 µm, *w*_*r*_ = 0.8 µm, *h*_*r*_ = 0.5 µm, *d* = 0.7 µm. We model the transmission in CST Studio, where we employ Floquet ports and periodic boundary conditions. The edge length of the square unit cell (*p*) is 1.5 µm. The polarization of the incident plane wave is shown in Figure 1(a).

Figure 1(b) illustrates the electric field distribution at the q-BIC resonance when the distance between the cuboids and bars (*a*) is 1 µm. Transmission and reflection spectra for various distances between the bars and cuboids are depicted in Figure 1(c) and (d). This structure enables out-of-band transmission of near than or below 0.1 within the 3,100–3,300 nm range when tuning *a* from 0.2 to 1 µm and less than 0.04 across 2,700–3,400 nm when *a* is set to 1 µm. Figure 1(c) shows only a part of the wavelength range to highlight the resonance features and their shift more clearly.

In the absence of cuboids, no q-BIC resonance appears, as the non-radiating BIC mode remains confined within the bars. Nevertheless, the BIC mode was identified using the eigenmode solver. Introducing asymmetry by positioning the cuboids above the bars enables radiation leakage, leading to the appearance of high-*Q* resonances in transmission.

We use the Fano formula [35], [36] to fit the transmission curves shown in Figure 1(c), enabling accurate analysis of the characteristics of the excited q-BIC resonances while accounting for their asymmetry:

$$T_{\text{Fano}}\left(\omega \right)=t_{d_{0}}+t_{d}^{2}\frac{{\left[q+\frac{2\left(\omega -\omega_{0}\right)}{d\omega }\right]}^{2}}{1+\frac{4{\left(\omega -\omega_{0}\right)}^{2}}{d\omega^{2}}},$$

where $t_{d_{0}}$ is the minimum of transmission near resonance, *t*_*d*_ is the non-resonant transmission amplitude, *q* is the shape factor, *ω*_0_ is the resonance frequency, *dω* is the resonance width.

**Figure 2:**
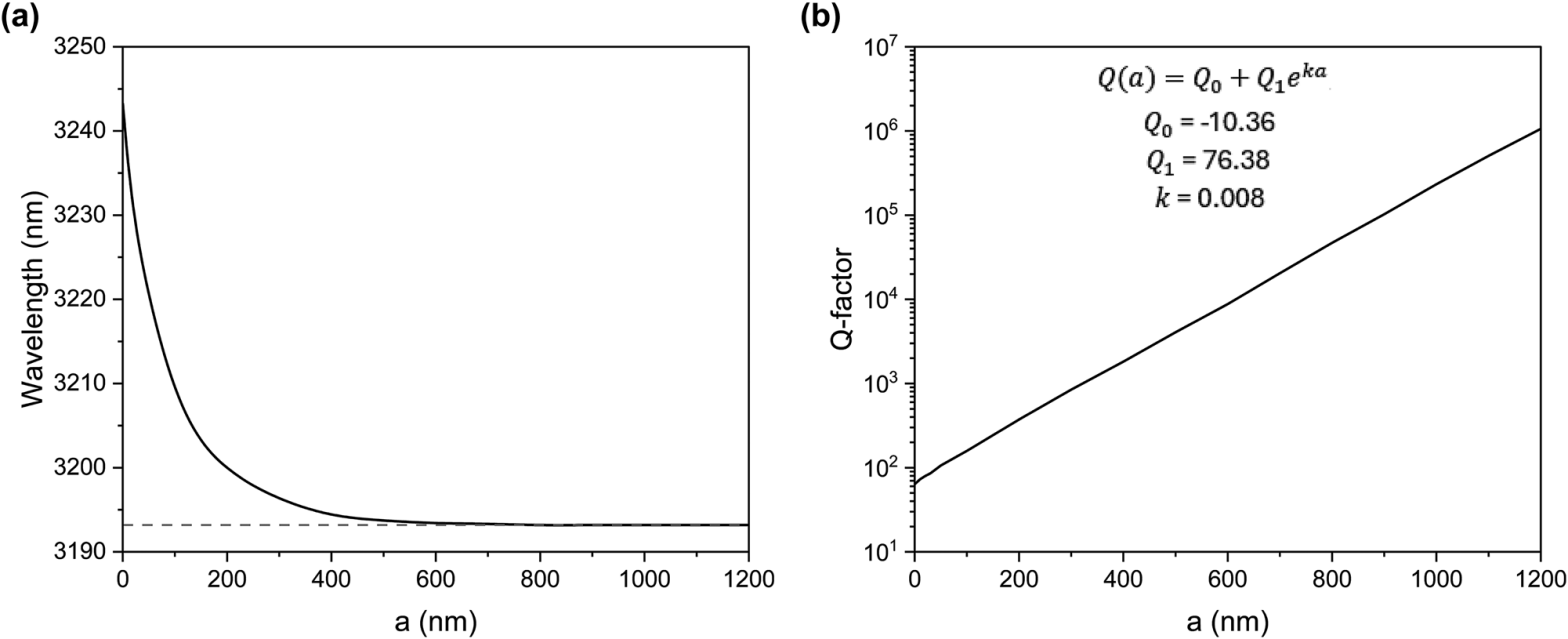
Evolution of the q-BIC resonance. (a) and (b) Dependence of the q-BIC resonance wavelength and *Q*-factor on the distance between the bars and cuboids.

Figure 2(a) and (b) show the dependence of the q-BIC resonance wavelength and the *Q*-factor on the distance between the bars and the cuboids. The q-BIC resonance wavelength approaches the BIC value of approximately 3,193.2 nm as the cuboids move away from the 1D metasurface. To accurately describe the relationship between the *Q*-factor of the excited q-BIC resonances and the distance between the cuboids and the bars depicted in Figure 2(b), we can use the exponential growth formula:

$$Q\left(a\right)=Q_{0}+Q_{1}e^{ka},$$

where *Q*_0_ is the offset parameter, *Q*_1_ is the initial *Q*-factor value, *k* is the rate. The curve has the following parameters: *Q*_0_ = −10.36, *Q*_1_ = 76.38 and *k* = 0.008.

Figure 2(b) shows that the *Q*-factor exponentially increases as the gap (*a*) widens, reaching a value of 10^6^ when the gap is 1.2 µm. This behavior confirms the q-BIC nature of the resonance, as the quality factor of the q-BIC mode theoretically approaches infinity with decreasing asymmetry. The proximity of the cuboids to the 1D metasurface introduces asymmetry into the field distribution excited by the incident wave. Conversely, increasing the distance between the cuboids and the 1D metasurface restores the system’s symmetry, thereby reducing radiation leakage. Such control over the *Q*-factor of the q-BIC resonances, realized through out-of-plane symmetry breaking, offers an additional degree of freedom for tuning their characteristics. Figure 1(c) and (d) illustrate that, as the *Q*-factor increases, the transmission level decreases while the reflection level increases. This behavior is also accompanied by higher losses at the resonance wavelength when approaching the BIC state. Additionally, the vertical movement of the cuboids does not allow significant tuning of the central wavelength unless the separation is reduced to less than 100 nm, as shown in Figures 1(c), (d) and 2(a).

**Figure 3:**
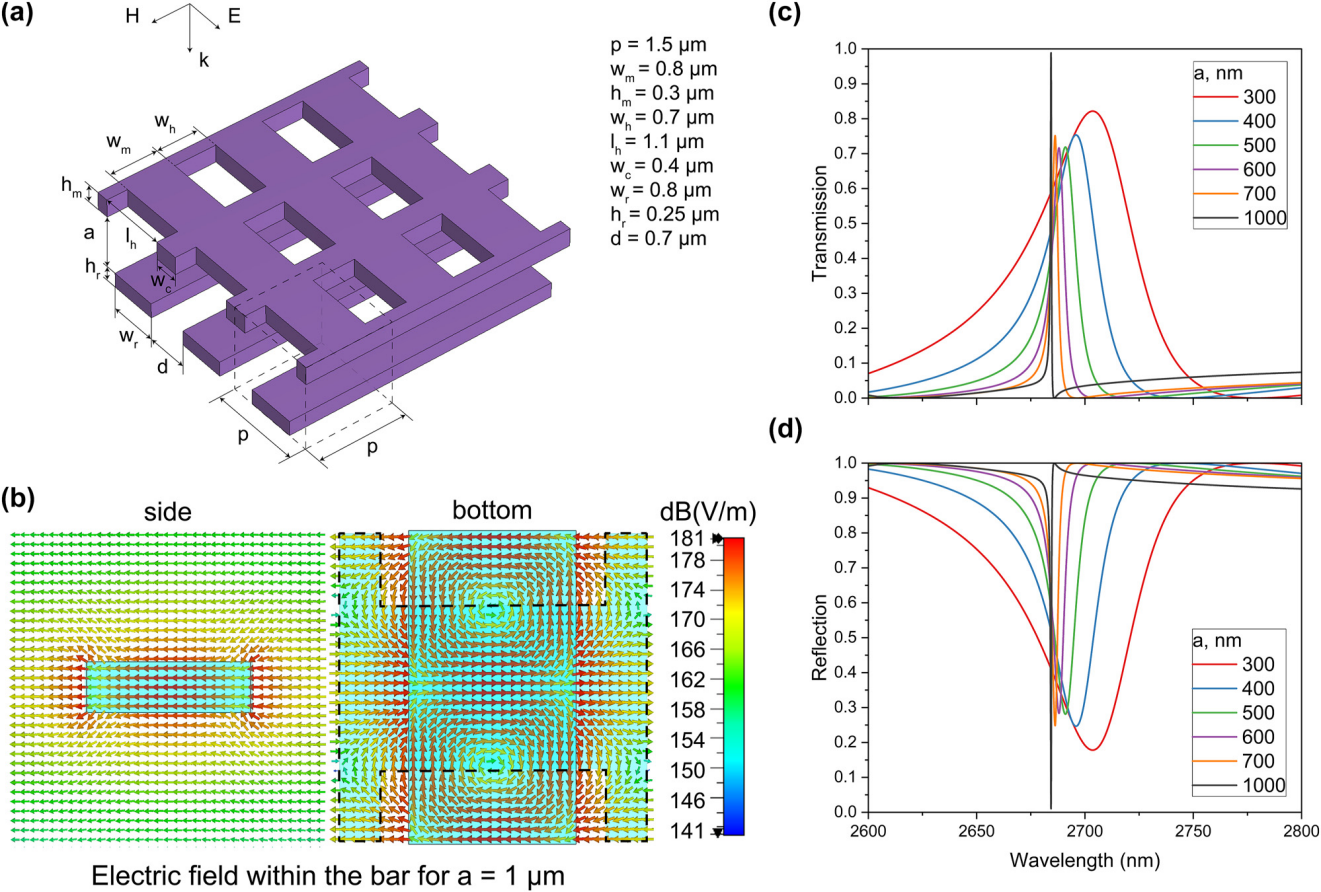
Metasurfaces based on the vertically adjustable membrane. (a) Schematic of the tunable metasurface design consisting of silicon bars and a MEMS membrane, including the unit cell indicated by the dashed lines; (b) maximum electric field amplitude of the q-BIC mode within the bar for *a* = 1 µm (side and bottom views, the dashed lines show the position of the MEMS membrane); (c) and (d) transmission and reflection coefficients for various distances between two layers (*a*).

Next, we examine a more practical design featuring a 1D array of bars and a 2D MEMS membrane, as schematically shown in Figure 3(a), which enables control of the same q-BIC resonance. The MEMS membrane is flat and thin, offering a significant advantage over bulk cuboids that would require attachment to a supporting scaffolding structure for manufacturing, thereby complicating the process. Using flat membranes instead of volumetric structures greatly simplifies fabrication.

Figure 3(a) illustrates the schematic of the proposed tunable metasurface design, comprising silicon bars and a perforated MEMS membrane, along with its transmission characteristics for varying distances between the two layers (*a*). The structural parameters are as follows: *w*_*m*_ = 0.8 µm, *h*_*m*_ = 0.3 µm, *w*_*h*_ = 0.7 µm, *l*_*h*_ = 1.1 µm, *w*_*c*_ = 0.4 µm, *w*_*r*_ = 0.8 µm, *h*_*r*_ = 0.25 µm, *d* = 0.7 µm. The edge length of the square unit cell (*p*) is 1.5 µm. The polarization of the incident plane wave is shown using the triple of vectors in Figure 3(a). Figure 3(b) demonstrates that this design enables the excitation of the q-BIC resonance, with the electric field distribution corresponding to the loops in the bars previously depicted in Figure 1(b).

Figure 3(c) and (d) illustrate that the vertical movement of the MEMS membrane enables control over the *Q*-factor of the q-BIC resonances but does not provide substantial tuning of the resonance peak wavelength. The q-BIC resonance shifts by less than 20 nm when *a* is varied from 0.3 to 1 µm, corresponding to less than 1 % of the resonance wavelength. The *Q*-factor also varies with *a*, and the resonance exhibits an ultranarrow bandwidth of approximately 1 nm when the distance between the layers is 0.9 µm. This indicates that the MEMS-based tuning method is effective for adjusting the bandwidth but is not suitable for applications requiring significant wavelength tunability.

The height of the bars was optimized to shift the q-BIC resonance into the range of maximum reflection. This adjustment accounts for the structural differences between the cuboids and the perforated MEMS membrane, achieving out-of-band transmission below 0.1 within the 2,600–2,900 nm range when *a* is tuned from 0.3 µm to 1 µm. Such performance makes the proposed system suitable for spectrometers employing broadband receivers in combination with tunable filters [21].

Interestingly, the peak transmission level does not decrease with increasing the *Q*-factor and *a*, suggesting a more complex behavior of the q-BIC resonance compared to the previous case with cuboids and bars, where the peak transmission decreased as *a* increased. While losses remain proportional to the *Q*-factor, Figure 3(c) shows that the reflection at the resonance wavelength does not increase with *a*. This observation highlights the potential for optimizing the 2D MEMS structure to maintain high transmission values at the resonance wavelength while achieving the desired ultranarrow bandwidth.

The bars and the overlying membrane can be attached to a supporting structure (not shown in Figure 3(a)). Alternatively, the bars may be placed on a quartz substrate, which can improve the mechanical stability of the proposed metasurfaces. However, this would also increase their thickness and limit the horizontal movement of the bars. As mentioned earlier, many tunable filter applications require maintaining a constant *Q*-factor while significantly shifting the resonance wavelength. To meet this requirement, we further investigate the horizontal movement of the bars to gain additional control over the resonance peak wavelength.

**Figure 4:**
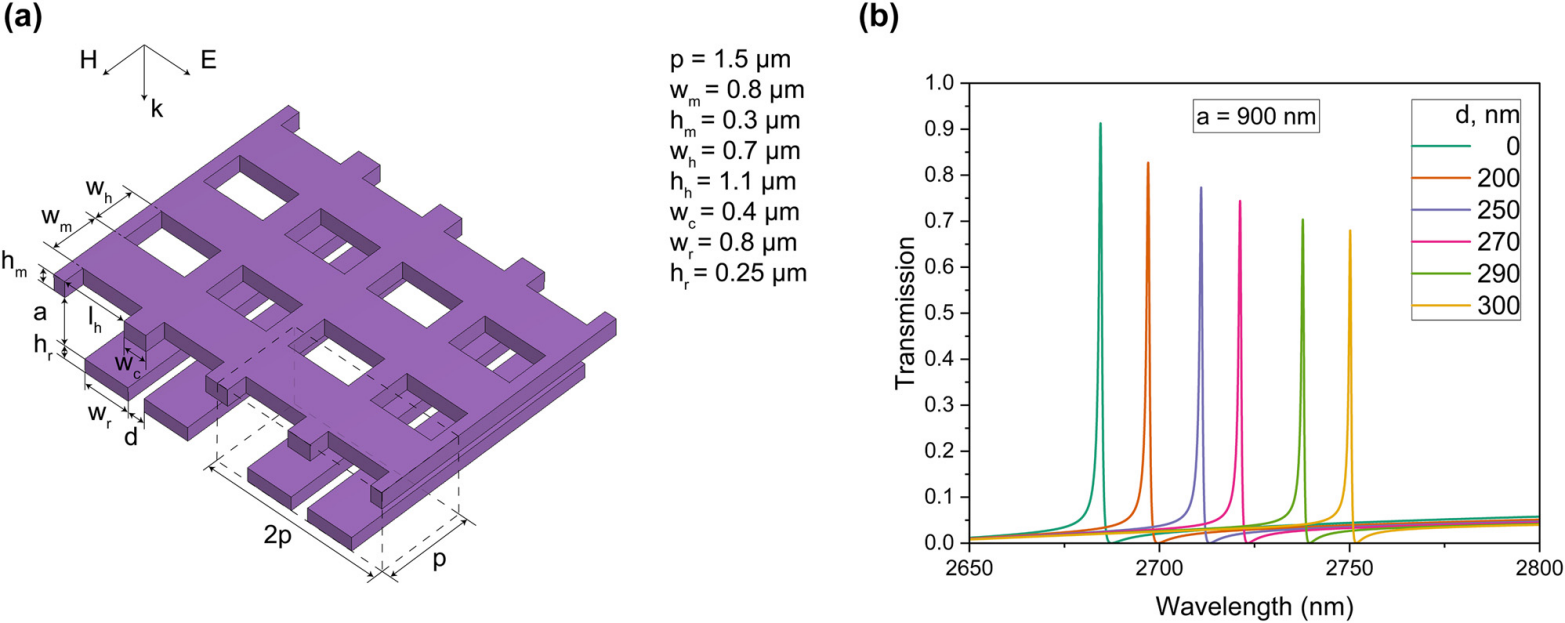
Metasurfaces based on two independent orthogonal tuning mechanisms. (a) Schematic of the tunable metasurface design consisting of pairs of silicon bars and a MEMS membrane, including the unit cell indicated by the dashed lines; (b) transmission characteristics for varying distances between the bars (*d*), with the distance between the two layers (*a*) fixed at 900 nm.

Figure 4(a) depicts the tunable metasurface design consisting of pairs of silicon bars that can move horizontally in the direction normal to their axis, while the membrane moves vertically above the bars. The structural parameters remain consistent with those in Figure 3(a), but we alter the distance between the silicon bar pairs (*d*), while keeping the period *p* constant. The polarization of the incident plane wave is shown in Figure 4(a). Two independent orthogonal tuning mechanisms enable precise control of both the central wavelength and bandwidth (or *Q*-factor) of the q-BIC resonance.

Figure 4(b) shows the transmission coefficient for various distances between the bars (*d*) when *a* = 900 nm. The horizontal movement of the bars allows for a resonance wavelength shift of approximately 65 nm. In this case, out-of-band transmission is less than 0.1 within the 2,600–3,000 nm range. The specular reflection remains almost constant as the distance between the bars increases. The reduction in transmission at the q-BIC resonance in Figure 4(b) arises from enhanced coupling of the incident wave into the first-order diffracted modes.

This approach alone does not maintain a constant bandwidth or *Q*-factor. As previously demonstrated, precise bandwidth control is achieved through the vertical movement of the membrane. By combining two independent orthogonal tuning mechanisms, complete control over the q-BIC resonance characteristics can be realized.

**Figure 5:**
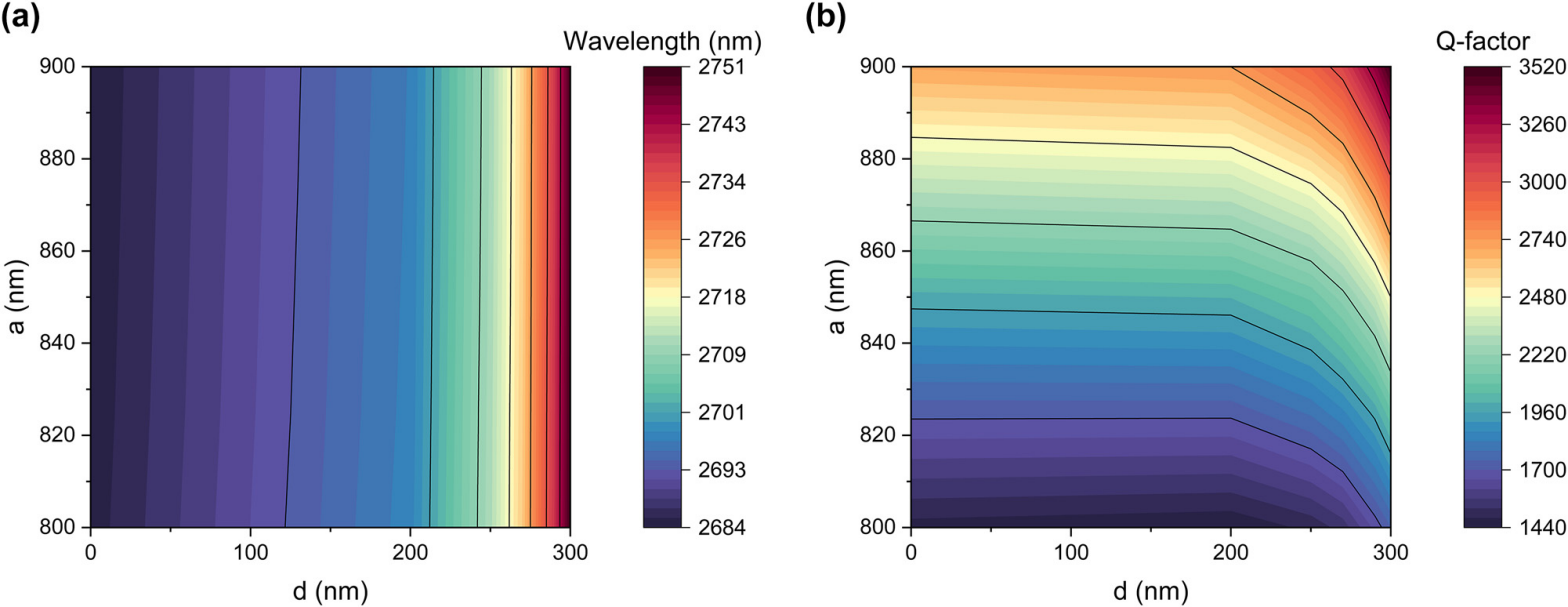
Tuning of the q-BIC resonance. (a) and (b) Dependence of the resonance central wavelength and *Q*-factor on two parameters: the distance between the two layers (*a*) and the distance between the bars (*d*). The black lines indicate examples of curves with constant wavelength and *Q*-factor, respectively.

Figure 5(a) and (b) illustrate the dependence of the resonance central wavelength and *Q*-factor on two parameters: the distance between two layers (*a*) and the distance between the bars (*d*). By precisely adjusting both parameters, as indicated by the black lines in Figure 5(b), it is possible to maintain a constant *Q*-factor while tuning the central wavelength by more than 60 nm.

Table 1 summarizes the characteristics of three investigated tunable metasurface designs:

(i) Metasurface concept utilizing vertically adjustable cuboids, as shown in Figure 1(a);
(ii) Metasurface employing a single tuning mechanism based on a vertically adjustable perforated membrane, as illustrated in Figure 3(a);
(iii) Metasurfaces incorporating two independent orthogonal tuning mechanisms, as depicted in Figure 4(a).

**Table 1:** Comparison of characteristics of the proposed tunable metasurfaces.

Characteristic	Metasurfaces based on vertically adjustable cuboids	Metasurfaces based on the vertically adjustable membrane	Metasurfaces based on two independent orthogonal tuning mechanisms
Spectral tuning range for *a* > 100 nm	15 nm	50 nm	65 nm
Constant *Q* factor	–	–	+
Range in which transmission is less than 10 % (for 1 nm resolution)	2,700–3,500 nm	2,600–3,000 nm	2,600–3,000 nm
Flat MEMS structure	–	+	+

Table 1 highlights the benefits of utilizing two independent orthogonal tuning mechanisms. These mechanisms enable the preservation of a consistent *Q*-factor of the q-BIC resonance while substantially extending the spectral tuning range. Additionally, the design ensures flat MEMS structures and achieves out-of-band transmission of less than 0.1 across a broad range near the q-BIC resonance.

## 3 Discussion

Our study introduces a new way to control q-BIC resonances in all-silicon metasurfaces. This is achieved by breaking out-of-plane symmetry with a supplementary 2D structure on top of a 1D metasurface where the q-BIC mode is excited. By changing the distance between the 2D MEMS structure and the 1D metasurface, we gain precise control over the *Q*-factor of the q-BIC resonance. Additionally, by combining two independent orthogonal tuning mechanisms within the proposed tunable metasurface designs, we demonstrated a spectral tuning range of more than 60 nm for the ultranarrow q-BIC resonance in transmission while maintaining a consistent *Q*-factor and out-of-band transmission below 0.1 across a broad range.

Our theoretical work outlines pathways for the experimental realization of sub-nm resolution filters combined with a wide tuning range, enabling advancements in spectroscopy and hyperspectral imaging. The proposed tunable metasurfaces can be fabricated using techniques similar to those employed for nano-electromechanical tunable suspended gratings [32]. In this approach, a buffered silicon oxide layer beneath a 1D metasurface is partially etched to achieve suspension, while the anchors remain supported by the oxide layer, ensuring structural stability.

However, we acknowledge that the experimental implementation of two independent orthogonal tuning mechanisms presents greater challenges. The bandwidth and *Q*-factor of the proposed metasurfaces are expected to be constrained by fabrication accuracy. Variations in the sizes of the bars, cuboids, or perforated holes in the membranes may lead to a broadening of the q-BIC resonances. Maintaining parallelism of nanostructured membranes at such small distances presents a significant practical challenge. Misalignments and membrane non-flatness are expected to broaden resonances in experiments. These detrimental effects can be mitigated by incorporating appropriate stress-compensation strategies. Counterbalancing stress-induced deformation in MEMS structures can be achieved by introducing additional layers with controlled thickness and intrinsic stress at strategic locations, thereby compensating for undesired deformations in a predictable manner [37]. Furthermore, the proposed tunable metasurface designs do not require MEMS membranes to approach the 1D metasurface closer than a few hundred nanometres, which greatly simplifies experimental realisation. Control of extraordinary optical transmission resonances through similar vertical displacement of MEMS membranes has recently been demonstrated for tunable filtering in the long-wavelength infrared range [38], showing that achieving near-zero air gaps is challenging due to fabrication non-uniformities, surface roughness, and the pull-in effect.

Our additional findings indicate that the horizontal movement of cuboids or membranes does not provide significant tunability of the q-BIC resonance wavelength while maintaining a 1 nm bandwidth. This suggests that achieving significant tunability of the q-BIC resonance wavelength within the studied metasurfaces requires modifications to the bars, where the maximum electric field is observed at resonance.

An alternative approach to realizing q-BIC resonances could be based on introduction of asymmetry within the meta-atom by positioning structures near one section of the meta-atom while leaving the other section unaffected by any additional layers. This method resembles in-plane symmetry breaking [39], [40], where geometric asymmetry enables resonance control. However, this approach will likely require MEMS structures to move close to 1D metasurfaces, which may pose challenges due to imperfections in the fabricated meta-structures.

Our work extends the exploration of additional degrees of freedom for manipulating the characteristics of q-BIC resonances and can be integrated with previously proposed permittivity-asymmetric methods [41] that rely on the electric control of silicon properties, as well as other approaches utilizing the thermal tuning of silicon [33]. MEMS structures that can be moved close to metasurfaces, particularly near high-symmetry points of undisturbed BIC modes, could act as perturbative elements and couplers to the radiation continuum [42]. The out-of-plane dimension can be leveraged to control optical chirality [43] and other key characteristics of metasurfaces, further enhancing the range of applications for mechanically tunable q-BIC resonances.

## 4 Conclusions

This study introduces a new method for controlling q-BIC resonances in all-silicon metasurfaces by using mechanical movements for dynamic tuning. Through breaking out-of-plane symmetry with perforated 2D MEMS membranes integrated above 1D metasurfaces, the proposed design achieves precise control over the wavelength and *Q*-factor of q-BIC resonances. Leveraging two independent orthogonal tuning mechanisms, the proposed method offers a spectral tuning range over 60 nm while keeping a stable *Q*-factor. This advancement has great potential for applications requiring tunable narrow resonances, such as imaging, sensing, and spectroscopy. Additionally, this dual MEMS tuning method can be applied to control other types of resonances.
